# Biocontrol of larval mosquitoes by *Acilius sulcatus *(Coleoptera: Dytiscidae)

**DOI:** 10.1186/1471-2334-8-138

**Published:** 2008-10-15

**Authors:** Goutam Chandra, Samir K Mandal, Arup K Ghosh, Dipanwita Das, Siddhartha S Banerjee, Sumanta Chakraborty

**Affiliations:** 1 Mosquito Research Unit, Department of Zoology, the University of Burdwan, West Bengal, India

## Abstract

**Background:**

Problems associated with resistant mosquitoes and the effects on non-target species by chemicals, evoke a reason to find alternative methods to control mosquitoes, like the use of natural predators. In this regard, aquatic coleopterans have been explored less compared to other insect predators. In the present study, an evaluation of the role of the larvae of *Acilius sulcatus *Linnaeus 1758 (Coleoptera: Dytiscidae) as predator of mosquito immatures was made in the laboratory. Its efficacy under field condition was also determined to emphasize its potential as bio-control agent of mosquitoes.

**Methods:**

In the laboratory, the predation potential of the larvae of *A. sulcatus *was assessed using the larvae of *Culex quinquefasciatus *Say 1823 (Diptera: Culicidae) as prey at varying predator and prey densities and available space. Under field conditions, the effectiveness of the larvae of *A. sulcatus *was evaluated through augmentative release in ten cemented tanks hosting immatures of different mosquito species at varying density. The dip density changes in the mosquito immatures were used as indicator for the effectiveness of *A. sulcatus *larvae.

**Results:**

A single larva of *A. sulcatus *consumed on an average 34 IV instar larvae of *Cx. quinquefasciatus *in a 24 h period. It was observed that feeding rate of *A. sulcatus *did not differ between the light-on (6 a.m. – 6 p.m.), and dark (6 p.m. – 6 a.m.) phases, but decreased with the volume of water i.e., space availability. The prey consumption of the larvae of *A. sulcatus *differed significantly (P < 0.05) with different prey, predator and volume combinations, revealed through univariate ANOVA. The field study revealed a significant decrease (p < 0.05) in larval density of different species of mosquitoes after 30 days from the introduction of *A. sulcatus *larvae, while with the withdrawal, a significant increase (p < 0.05) in larval density was noted indicating the efficacy of *A. sulcatus *in regulating mosquito immatures. In the control tanks, mean larval density did not differ (p > 0.05) throughout the study period.

**Conclusion:**

the larvae of the dytiscid beetle *A. sulcatus *proved to be an efficient predator of mosquito immatures and may be useful in biocontrol of medically important mosquitoes.

## Background

The chemical methods to regulate mosquito populations bear adverse impacts of resistant strains and effects on the non-target species [1,2]. As a sustainable alternative, increasing attention is being paid to control mosquitoes by biological means, including the utilization of natural predators of mosquito immatures [3,4]. Many of these predators, like the larvivorous fishes [5], crustaceans *Triops newberryii *[6] and *Mesoccyclops thermocyclopoides *[7], the belostomatid bugs *Diplonychus (= Sphaerodema) annulatus*, *D. rusticus *[8-11], notonectid bugs *Notonecta maculata *[12]*Enithares indica *[13], *Anisops bouvieri *[14,15] and the odonates like *Enallagma civile *[16], *Anax imperator *[17], *Brachytron pratense *[18] and some other species [19-23] have shown potential in regulating mosquito immatures. Even, the predatory larvae of the mosquitoes *Toxorhynchites rutilus *[24] and *Tx. splendens *[25] have been efficient in regulating vector and pest mosquito populations, both under field and laboratory conditions [26]. In smaller annual habitats like containers and tree holes, the copepods, especially, *Mesocyclops thermocyclopoides *[7,27], can be effective in regulating population of mosquito immatures.

Of the several predators stated above, coleopteran larvae are one such group that has been explored less compared to other similar controphic insects. The dytiscid beetles like *Rhantus sikkimensis *[28], *R. consputus *[29] and *Colymbates paykulli, Hydroporus *sp, and *Ilybus ater *[30] are known to prey upon mosquito immatures. In ricefields and temporary pools, larvae and adults of dytiscid beetles are dominant predators and have a strong impact in regulating tadpoles and different insect groups [31-34]. Considering the predatory nature of the dytiscid beetles, an assessment of the larvae of *Acilius sulcatus *Linnaeus 1758 (Coleoptera: Dytiscidae) was made as bio-control agent against the filarial vector *Culex quinquefasciatus *Say 1823, both under laboratory and field conditions. The dytiscid beetles *A. sulcatus *are common to abundant in the ricefield and temporary pool and bogs of West Bengal, India. Since these habitats are also exploited by mosquitoes fro breeding, the results of the present study will highlight their use in such situations as biocontrol agents.

## Methods

### Laboratory experiment

The IV instar larvae of *A. sulcatus *(Order: Coleoptera) were collected from shallow ponds and rice fields and Larvae of *Cx. quinquefasciatus *from drains of Burdwan, West Bengal, India and colony was maintained in the Mosquito Research Unit of the Department of Zoology, Burdwan University. Average length of *A. sulcatus *larvae taken for study was 1.7 cm (± 0.12 SE; n = 10). One and five *A. sulcatus *were provided with 200 and 1000 newly emerged IV instar *Cx. quinquefasciatus *larvae as prey for a period of 24 hr in glass jars (20 cm × 15 cm × 25 cm ; 5 L capacity) containing 1 L and 5 L of pond water respectively. The pond water of the habitat of *A. sulcatus*, was used in the experiments after sieving through a net (>500 mesh) to exclude any larvae of other predator species. The water temperature ranged from 23.8 to 27°C, pH from 6.67 to 6.84 and dissolved oxygen from 5.33 to 6.23 mg/l, during the experiment. Abrupt changes in the quality of holding water during rearing and experimentation were avoided. The predation experiment was conducted three times on three separate days, each with three replications. A control experiment was done every time. The number of *Cx. quinquefasciatus *larvae consumed by *A. sulcatus *larva during lights-on (0600 – 1800 h, IST-Indian Standard Time) and lights-off phase (1800 – 0600 h, IST) was noted through one day at an interval of 3 hrs. After counting the number of consumed larvae, after every 3 hr, the same numbers of larvae were replenished within the glass jars to maintain the same prey density. The experiment was commenced at 6 a.m. of a day and those were completed at 6 a.m. of the next day to observe the daily feeding rate. The length of the lights-on and lights-off phases were maintained by the application of artificial lights (Tube fluorescent lights), set on the walls of the laboratory (6 × 40 Watt). The lights-on phase in the laboratory synchronized manually with the natural outdoor sunlight photo phase and the lights-off phase with the dark phase of the night.

To evaluate the predation of the larval forms of *A. sulcatus *on the 4th instar larvae of *Cx. quinquefasciatus*, different combinations of prey, predator and volume of water were considered. In each case nine replicate for one combination were made. The combinations were as stated below:

a) 1 predator, 1 litre of water, 200 numbers of prey – combination A

b) 1 predator, 2 litres of water, 200 numbers of prey – combination B

c) 1 predator, 1 litre of water and 400 numbers of prey – combination C

d) 2 predators, 1 litre of water and 200 numbers of prey – combination D

e) 2 predators, 2 litre of water and 200 numbers of prey – combination E

f) 2 predators, 1 litre of water and 400 numbers of prey – combination F

The rate of predation for a period of 24 h was noted and the data was used to calculate the clearance rate (CR = number of prey/h/predator) an indicator of predatory efficiency. The CR values were obtained applying the following formula, following Gilbert and Burns [35], with certain modifications;

**CR = V.(lnP)/T.N**

Where, V = volume of water (in litre), ln = Natural log, P = number of prey killed/consumed, T = time, i.e. 24 h., N = number of predator.

### Field experiment

To examine the efficacy of larvae of *A. sulcatus *in field condition, Sainthia in the district of Birbhum, West Bengal, India was selected. In the study area, ten cemented tanks (which were located in open air and contained about 250 L of water) were selected. These tanks are usually used in processing of paddy. The tanks remain unused for a long time i.e. from post rainy season to early spring (Sep to Feb), and played the role as natural breeding places of mosquitoes. The tanks were made free from any larvae or nymphs of larvivorous insects or fishes by a fine net having mesh size of >130, which allowed the passage of mosquito larvae. Those tanks contained mosquito larvae of different species namely *Cx. quinquefasciatus *(Say,1823), *Cx. bitaeniorhynchus *(Giles,1901), *Cx. tritaeniorhynchus *(Giles, 1901), *Cx. vishnui *(Theobald, 1901), *Cx. gelidus *(Theobald,1901), *An. subpictus *(Grassi, 1889), *An. vagus *(Doenitz, 1902), *An. aconitus *(Doenitz,1902), *An. barbirostris *(Van der Wulp, 1884), *An. annularis *(Van der Wulp, 1884) and *Armigeres subalbatus *(Coquillett,1898). Each time 5 dips were taken in each tank and the mean per dip (250 ml dipper) larval density of each of those 10 tanks was assessed according to WHO, 1975 [36] for 15 times at an interval of 90 minutes on a specific day (total 5 × 15 dips in each tank). Then twenty larvae of *A. sulcatus *were introduced in each of first five tanks (No. 1 to 5). Five tanks no. 6 to 10 were kept as control to rule out the possibility of decreasing prey density by the activity of Zooplanktons like copepods. Larval densities in those tanks were assessed 30 days after introduction of *Acilius *larvae for 15 times in similar manner and on the next day all the predator larvae were removed. Densities were assessed again after 30 days from the withdrawal of *A. sulcatus *larvae from the tanks. The experiments (both in laboratory and in the field) were conducted in the months of June, July and August 2007. Re-colonization of tanks by larvae of other larvivorous insects was controlled by netting (>130 mesh) the tanks at an interval of 15 days during the study period. Methods of Ghosh *et al*. [37-39] and Chatterjee *et al*. [18] were used to conduct laboratory and field experiments during the present study.

The data obtained on clearance rate for each combination was subjected to one-way ANOVA followed by Tukey's post hoc test [40] to justify the differences, if any, between the combinations. Besides, Students' t-tests and 'Z' tests [40] were performed to evaluate the difference in mosquito larval density in the field conditions before and after the presence of the *A. sulcatus *larvae in the mesocosms.

## Results

### Laboratory experiment

**Figure 1:**
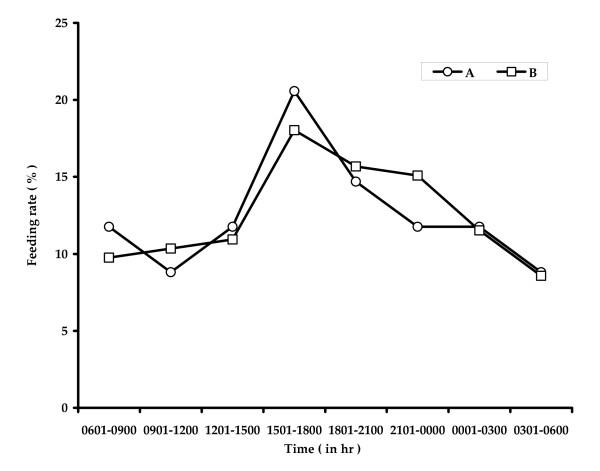
**Three-hourly feeding rate of larvae of *A. sulcatus *on IV instar larvae of *Cx. quinquefasciatus*.** A: 200 preys: 1 predator; B: 1000 preys: 5 predators.

**Figure 2:**
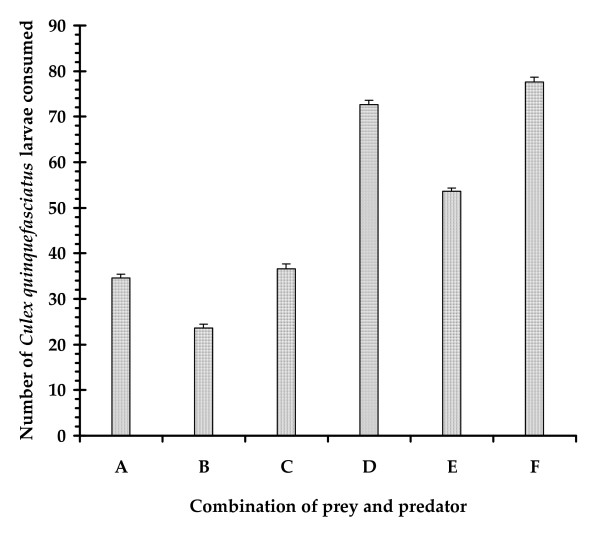
The number of prey consumed by a larva of *A. sulcatus *under different combinations (A – F) of prey, predator and volume of water.

**Figure 3:**
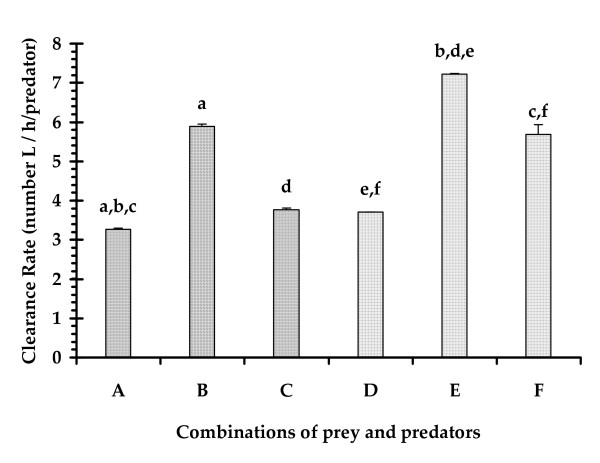
**The clearance rate of the larvae of *A. sulcatus *under different combinations (A – F) of prey, predator and water volume (n = 9 trials/combinations).** The letters shared by the bars represents significant differences between the combinations at P < 0.01, revealed through *post hoc *Tukey test.

The larva of *A. sulcatus *was observed to capture the mosquito larva prey on its head, sucked its body fluid as a part of extra oral digestion [34] and discarded some portions of the prey body within a few minutes. The feeding of the larval dytiscid beetles involves mandibles for capturing prey and transfer of enzymes into the tissues and accumulating partially digested pieces of the tissue [34]. The feeding posture of an *A. sulcatus *larva on a *Culex *larva has been presented in Plate [see Additional file 1]. Three hourly consumption rates of one and five *A. sulcatus *larvae on 200 and 1000 4^th ^instar *Cx. quinquefasciatus *larvae respectively have been presented in Fig 1. One *A. sulcatus *larva consumed 18 and 16 mosquito larvae during light on and light off phases respectively with a daily feeding rate of 34 larvae on an average. Five *A. sulcatus *larvae consumed 166 mosquito larvae during light on phase and 172 larvae during light off phase with a an average feeding rate of 33.8 larvae/predator in a 24 h period. The number of prey killed varied with the density of preys and predators available in a particular volume of water (Fig. 2). The clearance rate value ranged between 13.59 and 20.09 no. of prey L/h/predator. The maximum CR value was obtained when the predator larva was present in larger space (Fig. 3.). One way ANOVA revealed significant differences in the clearance rate at different combinations (Table 1). The result of the *post hoc *Tukey's test is presented in Fig 3. The results are suggestive of the fact that the larval stages of *A. sulcatus *exhibited varied predatory efficiency depending on the availability of space and the number of predators.

**Table 1:** Results of ANOVA on the clearance rate of *A. sulcatus *on IV instar *Cx. quinquefasciatus *larvae, under different combinations

Source of variation	SS	df	MS	F-value
Between combinations	4.68	5	0.94	9.14 (P < 0.001)
Residual	4.92	48	0.1	

Total	9.59	53		

### Field experiment

**Figure 4:**
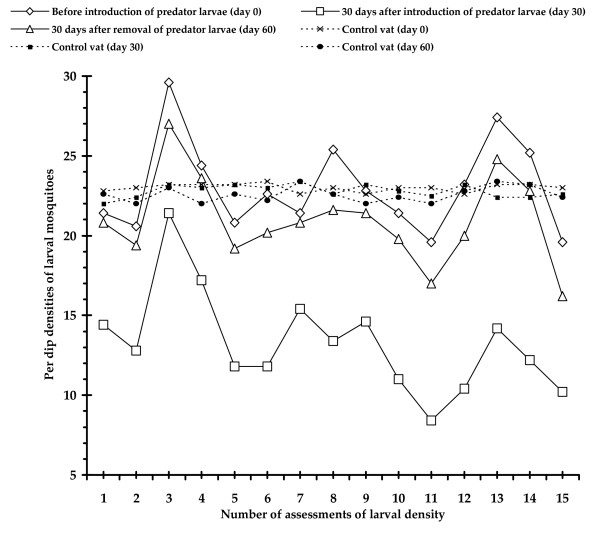
Efficacy of *A. sulcatus *larva as biocontrol agent against larval form of different mosquitoes in the field conditions.

Variations in the density of mosquito larval population with the introduction and removal of *A. sulcatus *larvae in the field are presented in Fig 4 which revealed that average per dip density of mosquito larvae reduced from 23.03 to 13.28 after 30 days from the introduction of *A. sulcatus *larvae in treated vats i.e., from vat no. 1 to 5 where the difference was significant (t_(0.05,14) _= 16.00 ; p < 0.05). Again larval density increased significantly (t_(0.05,14) _= 17.35 ; p < 0.05) from 13.28 to 20.97 in those vats (no. 1 to 5) after 30 days from the removal of predator species. Control vats (No. 6 to 10) did not show any difference (t_(0.05,14) _= 1.53 ; t_(0.05,14) _= 1.35; p > 0.05) in average larval densities (23.00, 22.79 and 22.57) throughout the study period [Table value of 't' = 2.145].

No predator mortality was recorded during the study period i.e. all 20 larvae of *A. sulcatus *introduced in each tank were actually recovered.

## Discussion

Present study revealed that *A. sulcatus *larva was active feeder and remained active throughout day and night, though it consumed apparently greater [Z = 1.233 (Table value = 1.56); p > 0.05] number of mosquito larvae during light on phase in comparison to light off phase. In recent years, predation and population regulation of *Culex *larvae by the dytiscid beetles *Rhantus sikkimensis *in India [28]*Hydroporus *sp, *Colymbetes paykulli *and *Ilybus ater *in Sweden [30]. However, under presence of alternative preys the selection for mosquito by these dytiscid beetles was less prominent compared to *R. consputus *[29]. In comparison to these beetles, the prey consumption of the larva of *A. sulcatus *was found to be higher. Further, under field conditions, the reduction in the populations of mosquito immatures in presence of the larvae of *A. sulcatus *indicates its efficiency as biocontrol agent. The efficacy of *A. sulcatus *was noted to be as good as larvivorous fishes like *Xenentodon cancila *fry, *Gambusia affinis *and *Poecilia reticulata *[41-43].

In the field larval densities reduced gradually after introduction of predator larvae in the mosquito breeding places and a significant difference in larval density was noted after one month from the introduction. On the other hand, larval densities increased gradually after removal of predator larvae from the treated breeding spots and a significant difference in larval density was noted after one month from their removal. Insignificant difference in larval density in the control tanks during the period of field experiment excluded the possibility of influence/effect of other factors and confirmed the role of predator in decreasing the larval density of mosquito in treated tanks. Considering the natural habitats of *A. sulcatus *and other aquatic coleopteran insects, the effects of alternative prey on the prey selection and predation ecology need to be evaluated prior to promoting these beetles for biological control. Like the dytiscid beetles *R. consputus *and the copepods *M. thermocyclopoides *positive selection for the mosquito larvae by *A. sulcatus *need to be evaluated. Nonetheless, in conditions of coexistence of multiple species of mosquitoes the larvae of *A. sulcatus *consumed the mosquito immatures without discrimination of a particular species. This is relevant in field conditions where multiple species of mosquitoes will be present.

## Conclusion

The *A. sulcatus *larva has been effective as predator of mosquito immatures and may be useful in biocontrol of medically important mosquitoes. From the viewpoint of efficient and sustainable biological control in the field condition, the aquatic predators should have a wide range of adaptability in the habitats apart from the predation of target mosquito larvae. Further work is necessary to determine the proper methodology of mass rearing and augmentative release of *Acilius *larva to make this biocontrol procedure possible for wide application.

## Competing interests

The authors declare that they have no competing interests.

## Authors' contributions

GC designed the study, supervised the experiments and wrote the manuscript in most parts. SKM performed the laboratory experiments. AKG, DD and SB performed the field experiments and data analysis. SC did statistical analysis and photography.

## Pre-publication history

The pre-publication history for this paper can be accessed here:



## Supplementary Material

Additional File 1 **Feeding posture of *A. sulcatus *on *Cx. quinquefasciatus *larvae.** Prey (*Cx. quinquefasciatus*) capture by the larva of *A. sulcatus*. Click here for file
