# Supplementary figures and images for: Biocontrol of larval mosquitoes by Acilius sulcatus (Coleoptera: Dytiscidae)
Source: BMC Infect Dis. 2008 Oct 15;8:138. doi: 10.1186/1471-2334-8-138 (PMC2573887; doi:10.1186/1471-2334-8-138)

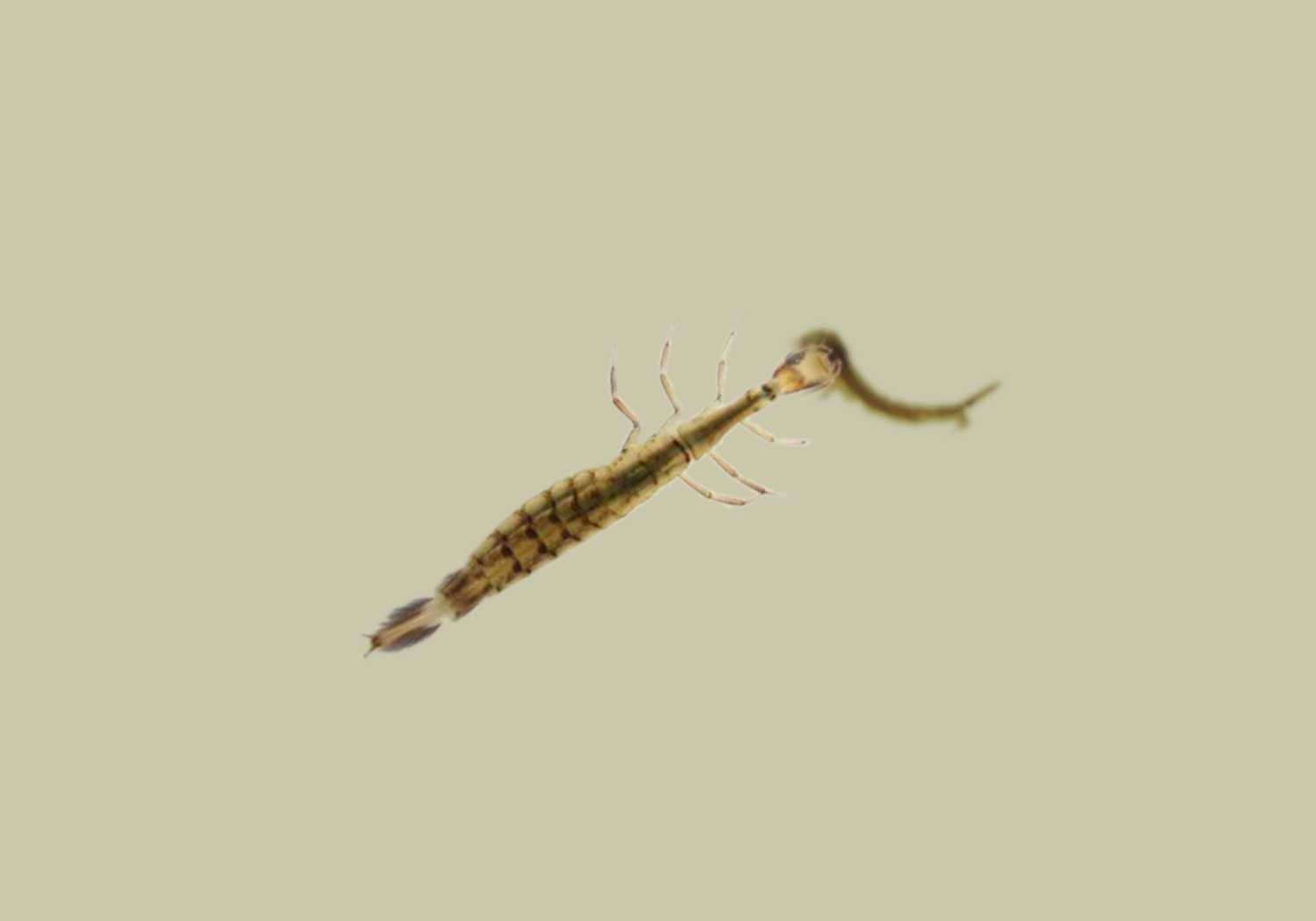

Supplement: Additional File 1 — Feeding posture of A. sulcatus on Cx. quinquefasciatus larvae. Prey (Cx. quinquefasciatus) capture by the larva of A. sulcatus. [file 1471-2334-8-138-S1.doc]
